# Psychosocial Determinants of Vegetable Intake Among Nepalese Young Adults: An Exploratory Survey

**DOI:** 10.3389/fnut.2021.688059

**Published:** 2021-06-10

**Authors:** Sujita Pandey, Mausam Budhathoki, Dipendra Kumar Yadav

**Affiliations:** ^1^Department of Food Science, University of Copenhagen, Frederiksberg, Denmark; ^2^Department of Management, Xavier International College, Kathmandu, Nepal; ^3^Faculty of Health Sciences, School of Health and Allied Sciences, Pokhara University, Pokhara, Nepal

**Keywords:** vegetable intake, psychosocial determinants, partial least square structural equation modelling, young adult, Nepal

## Abstract

**Background:** Adequate intake of vegetables facilitates a healthy lifestyle. However, the majority of Nepalese young adults consume inadequate amount of vegetables per day.

**Objectives:** We explored psychosocial determinants of daily intake of two or more servings of vegetables among Nepalese young adults using attitude, social influence, and self-efficacy (ASE) as a theoretical framework, extended with measures of habit and self-identity as additional constructs.

**Methods/Participants:** A cross-sectional study through a web-based questionnaire survey was conducted among 461 Nepalese young adults aged 18–35 years old. Participants were recruited through convenience (snowball) sampling. A factor-based partial least square structural equation modelling was used for analysis.

**Results:** The findings indicated that attitudes (β = 0.09, *p* = 0.029), social influence (β = 0.17, *p* < 0.001), habit (β = 0.24, *p* < 0.001) and self-identity (β = 0.30, *p* < 0.001) were significant factors influencing intention to eat two or more servings of vegetables per day. Further, self-efficacy (β = 0.10, *p* = 0.011), habit (β = 0.08, *p* = 0.034), diet (β = −0.10, *p* = 0.014), and place of residence (β = 0.11, *p* = 0.007) significantly influenced behaviour to eat two or more servings of vegetables per day. However, self-efficacy (β = 0.07, *p* = 0.062) did not significantly influence intention and self-identity (β = 0.06, *p* = 0.083), age (β = −0.02, *p* = 0.328), gender (β = 0.02, *p* = 0.350), and body mass index (β = −0.04, *p* = 0.209) did not significantly influence behaviour to eat two or more servings of vegetables per day.

**Conclusion:** The study shows that attitudes, social influence, habit, and self-identity were significant factors influencing intention to eat two or more servings of vegetables per day. Further, self-efficacy and habit significantly influenced behaviour to eat two or more servings of vegetables per day. However, self-efficacy did not significantly influence intention and self-identity did not significantly influence behaviour to eat two or more servings of vegetables per day.

## Introduction

Adequate intake of fruits and vegetables facilitates a healthy lifestyle. Several previous studies have linked consumption of inadequate fruits and vegetables to the growing burden of chronic diseases globally, such as gastrointestinal cancer (1), coronary heart disease (2), obesity (3), diabetes mellitus (4, 5), and stroke (6). The World Health Organisation estimates that ~1.7 million deaths globally are attributable to inadequate consumption of fruits and vegetables. Therefore, the report of a joint World Health Organisation and Food and Agriculture Organisation consultation (7) recommends consuming a minimum of 400 g of fruits and vegetables per day (excluding potatoes and other starchy tubers) to prevent these deaths due to chronic diseases and to mitigate micronutrient deficiencies. Further, consuming a variety of fruits and vegetables helps to ensure an adequate intake of many essential nutrients including, vitamins and minerals, dietary fibres and antioxidants (7). The recommended serving size of fruits and vegetables in Nepal is indicated descriptively, where the Ministry of Health and Population of Nepal recommends eating plenty of fruits and vegetables, especially green leafy vegetables.

In Nepal, the change in food culture in recent decades due to globalisation and urbanisation has resulted in unhealthy food habits among adolescents and young adults (8, 9). A nationwide STEPS survey (2013) results indicated that 99% of the 972 respondents aged 15–29 years old consumed insufficient quantities of fruits and vegetables (9). Recent survey data found that 76% of 1,600 young adults aged 18–29 years residing in the capital city Kathmandu consumed insufficient quantities of fruits and vegetables (10). On average, Nepalese males between the ages of 25 and 34 years old consume 1.45 ± 0.64 servings of vegetable, while females consume an average of 1.47 ± 0.62 servings of vegetables (11). It is important for young adults to consume adequate amount of vegetables because individual dietary patterns developed in young adults often persist into later life, influencing their own health as well as that of their partners and children (12, 13).

To our knowledge, no research has been conducted in developing countries like Nepal to determine psychosocial determinants of vegetable intake among young adults. Existing data have investigated only the sociodemographic and diet-related factors. Thus, the underlying psychosocial determinants remain unclear, and more research is needed to determine the key psychosocial determinants of behaviour regarding vegetable intake and their relationships to daily intake. The main aim of this study is to determine the psychosocial determinants influencing daily intake of two or more servings of vegetables among Nepalese young adults using attitude, social influence and self-efficacy (ASE) as a theoretical framework, extended with measures of habit, and self-identity as additional constructs. The findings from this study may support the development of effective intervention programmes for increasing vegetable intake among young adults in Nepal.

## Theoretical Framework

The ASE framework (14, 15) has been used extensively to study dietary behaviours (16, 17), including vegetable intake (17–19). According to the ASE model, social cognitive factors, namely attitudes, social influence and self-efficacy expectations are major determinants of health-related behaviour (20, 21). Further, behavioural intention acts as a mediator between the influence of attitudes, social norms and self-efficacy and health-related behaviour (22). The study (23) found that besides intention, Bandura's self-efficacy (24) was the most consistent factor explaining vegetable intake behaviour. Moreover, dietary behaviours are repeatedly and routinely performed and thus may eventually become habitual (25). Lally and Gardner (26) argue that habitual behaviours are automatic responses to environmental cues rather than a conscious decision process as proposed by the ASE framework. Studies have indicated that when habit strength increases, deliberate intentions of behaviour are less predictive (27, 28). The study by Brug et al. (17) found that habit is one of the major psychosocial determinants of fruit and vegetable consumption among adults and could be included as an independent determinant in health behaviour models. Further, previous studies have found that when habit is added as an additional construct to the health behaviour model, the predictive power of the model increased (25, 29–31). For instance, the study (31) which predicted fruit consumption showed that the addition of habit in the health behaviour model increased the explained variance up to 9%.

In addition to habit, self-identity—a salient aspect where a person recognises their self-perception and creates ideal decisions to act—is a motivational construct that makes a person more willing to perform health behaviour through influencing the behavioural intention (32, 33). Previous studies have shown that when a person recognises themselves as a healthy eater, the person will have positive effects on intention to eat fruits and vegetables (34–36). A meta-analysis by Rise et al. (37) found that self-identity as an additional construct in health behaviour models increased about 6% of variance in behavioural intention.

**Figure 1:**
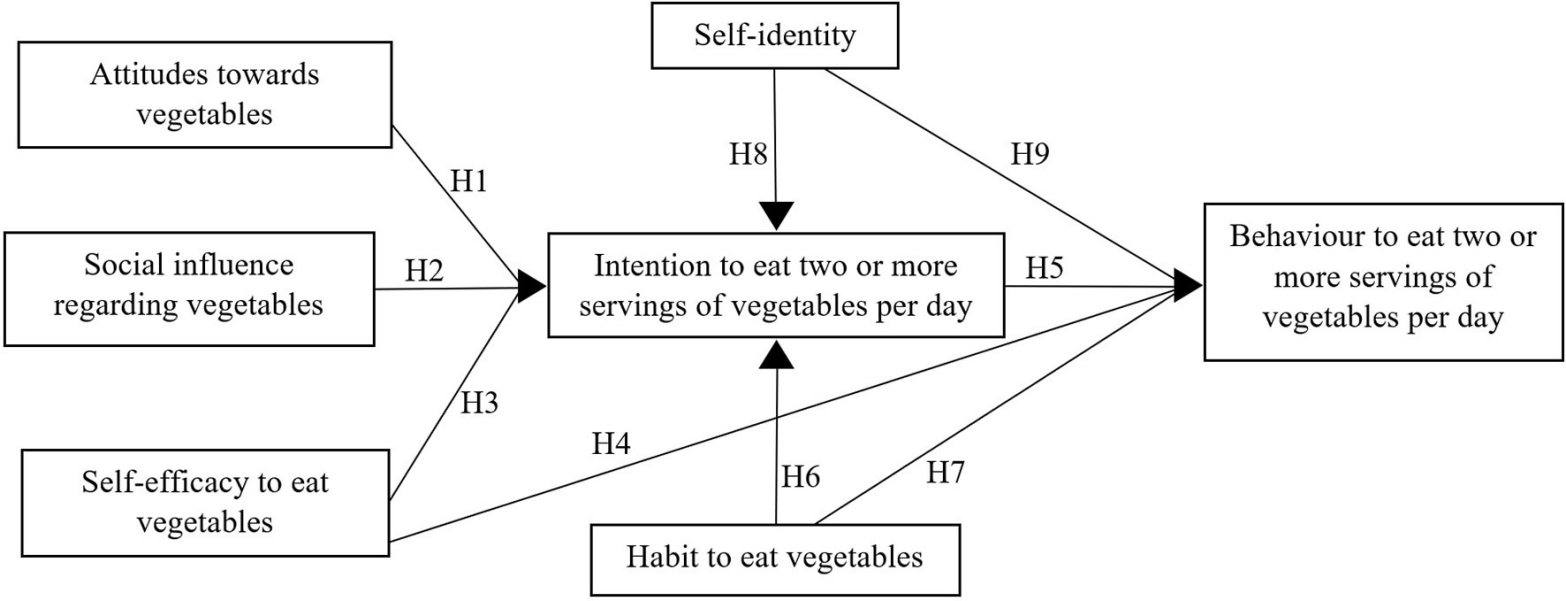
Proposed theoretical framework for determining daily intake of two or more servings of vegetables.

Based on the findings, following hypotheses were expected (see Figure 1):

Hypotheses 1–5: Attitudes, social influence, self-efficacy, and intention affect behaviour to eat two or more servings of vegetables per day.
Hypotheses 6–7: Habit to eat vegetables significantly affects intention as well as behaviour to eat two or more servings of vegetables per day.
Hypotheses 8–9: Self-identity significantly affects intentions as well as behaviour to eat two or more servings of vegetables per day.

## Materials and Methods

### Questionnaire and Measurement Scale

The study questionnaire consists of standardised and validated questions adopted from previous literatures. Supplementary Table 1 briefly presents the questions measuring the corresponding constructs of the proposed theoretical framework and their source of adoption. A seven-point Likert scale ranging from (−3): Strongly disagree to (+3): Strongly agree was used to evaluate the items measuring ASE constructs as well as the additional constructs of habit and self-identity except for behaviour. Behaviour to eat two or more servings of vegetables per day was measured with the following two items: (1) the frequency of vegetable items (cauliflower, lentils, spinach, tomato, etc.) consumed during the last 24 h ranging from 1 = “none” to 4 = “four or more servings” and (2) the number of servings of vegetables consumed last week ranging from 1 = “ <3 per week” to 6 = “more than 3 per day.” To determine the serving size a visual aid was provided where potatoes, sweet potatoes and other starchy roots were not classified as vegetables. Portion size for cooked vegetables and salads could be answered either in serving cups or in grams (1/2 serving cup cooked = 1 serving cup raw = 75 g, according to the Australian Dietary Guidelines). Sociodemographic characteristics which were considered are age, gender, height and weight, dietary pattern and place of residence.

### Participants and Procedure

According to Kock and Hadaya (38), the minimum required sample size for the partial least square structural equation method to estimate a 0.170 path coefficient at significance level 0.05 with a power of 0.980, is 474 based on the inverse square root method and 454 based on the gamma-exponential method. Thus, a snowball sampling technique was utilised to recruit 475 participants via social media (Facebook) and the researchers' own networks (a link was sent by email) on 24th February 2021 to 8th March 2021. The questionnaire was developed in and administered through the Survey-Xact platform. The inclusion criteria for participation in the survey were the age range 18–35 years old, English language skills and willingness to participate. Participants younger than 18 years of age and older than 35 years of age, without English language skills and unwillingness to participate were excluded from the study. Participants were made aware about the time needed (~5–8 min) before completing the survey.

### Ethical Consideration

This study was conducted in accordance with the Declaration of Helsinki. All procedures involving study participants were approved by the Institutional Ethical Review Committee of Nepal Health Research Council (Reg nr. 67/2021 P). Written informed consent was obtained from all participants before participating in the survey.

### Data Analysis

The survey data stored on the Survey-Xact platform was transferred to a file compatible with IBM SPSS Statistics version 26.0 to perform statistical analysis. Initially, a descriptive analysis was conducted for the sociodemographic characteristics of the participants. Data were presented with percentage and number for categorical variables and median and interquartile range for continuous variable. The two items measuring behaviour were transferred into a dichotomous variable with intake of vegetables fewer than two servings per day coded as 0 and two or more servings of vegetables intake per day coded as 1. Further, the two reversed statements measuring attitudes were recorded in the same direction. Body mass index (BMI) was calculated as weight (kg)/height (m^2^) and categorised using Asian specific BMI cut-off values underweight was defined as <18.5 kg/m^2^, normal weight as 18.5–22.99 kg/m^2^, overweight as 23–27.49 kg/m^2^, and obese ≥27.5 kg/m^2^ (39).

Secondly, factor loadings, validity (both convergent and discriminant), reliability and multicollinearity of the constructs and correlation coefficient between the constructs were determined in conjunction with SEM analysis in WrapPLS version 7.0 software (40). A factor-based partial least square structural equation modelling (PLSF-SEM) algorithm routine was selected for testing hypotheses H1–H9. The outer model analysis (PLSF type CFM3, which employs both loadings and reliabilities from Dijkstra's consistent PLS technique to estimate measurement error and true composite weights), the inner model analysis (the default Wrap 3 algorithm) and the Stable 3 resampling method were utilised (41, 42). The underlying assumption of the model was based on the original ASE framework (i.e., a direct path from attitude, social influence and self-efficacy towards behavioural intention). Analysis of the original ASE framework and the proposed theoretical framework was conducted to test whether the proposed theoretical model fits the data more accurately. The model fit was reported by the four goodness-of-fit measures: average path coefficient (APC), average r-squared (ARS) values, average variance inflation factors (AVIF), and the goodness-of-fit (GoF) global index.

## Results

### Sociodemographic Characteristics

Table 1 presents the sociodemographic characteristics of the participants. After excluding 28 incomplete questionnaires, the final sample consisted of 461 young adults between the age range of 18–35 years old, 56.0% of which were females. The result also indicated that the majority of the participants followed a non-vegetarian dietary pattern (76.4% of the participants), were from the capital city Kathmandu (42.1%) and had a normal BMI (kg/m^2^) (54.9%).

**Table 1:** Sociodemographic characteristics of the participants, *N* = 461^a^.

**Sociodemographic characteristics**	**Categories**	
Age, year, median (IQR)		22 (5)
Gender, % (*n*)	Male	44.0 (203)
	Female	56.0 (258)
Dietary pattern^b^, % (*n*)	Vegetarian	20.4 (94)
	Vegan	1.5 (7)
	Non-vegetarian	76.4 (352)
	Flexitarian	1.7 (8)
Place of residence, % (*n*)	Kathmandu	42.1 (194)
	Lalitpur	14.2 (65)
	Bhaktapur	21.0 (97)
	Outside Kathmandu Valley	22.8 (105)
BMI (kg/m^2^), % (*n*)	Below 18.5 (Underweight)	18.0 (83)
	18.5–22.99 (Normal weight)	54.9 (253)
	23.0–27.49 (Overweight)	18.4 (85)
	27.5 and above (Obese)	8.7 (40)

a *Data is presented as median (interquartile range, IQR) or percentage (frequency)*;

b *Dietary pattern: vegetarian avoids meat (and fish), but eats eggs and dairy products, vegan avoids all animal-based products, non-vegetarian eats everything, flexitarian is mainly vegetarian (with occasional meat consumption). Source: Own elaboration*.

### Confirmatory Factor Analysis, Validity, Reliability, and Multicollinearity Tests

The results from Supplementary Table 2 show that all items measuring the ASE constructs and additional constructs of habit and self-identity loaded highly on the pre-determined factors—normalised loadings for the items of each construct were above 0.70 (40, 43). Further, the *p*-values associated with the loadings of items in their corresponding constructs were <0.05, indicating acceptable convergent validity (42). The value of Cronbach's alpha and composite reliability, which assesses measurement reliability, were all above the minimum requirement of 0.70 (44). The full collinearity variance inflation factors (VIFs) of all constructs were <3.3, which suggests that no multicollinearity and no common method bias exists in the model (40). The value of average variance extracted (AVE) of each construct was above the minimum threshold of 0.50, as shown in Supplementary Table 2, and the square roots of the AVE were greater than the correlation coefficient among the constructs, as shown in Supplementary Table 3, which confirmed the discriminant validity of the constructs (45, 46).

### Goodness-of-Fit Measures

**Figure 2:**
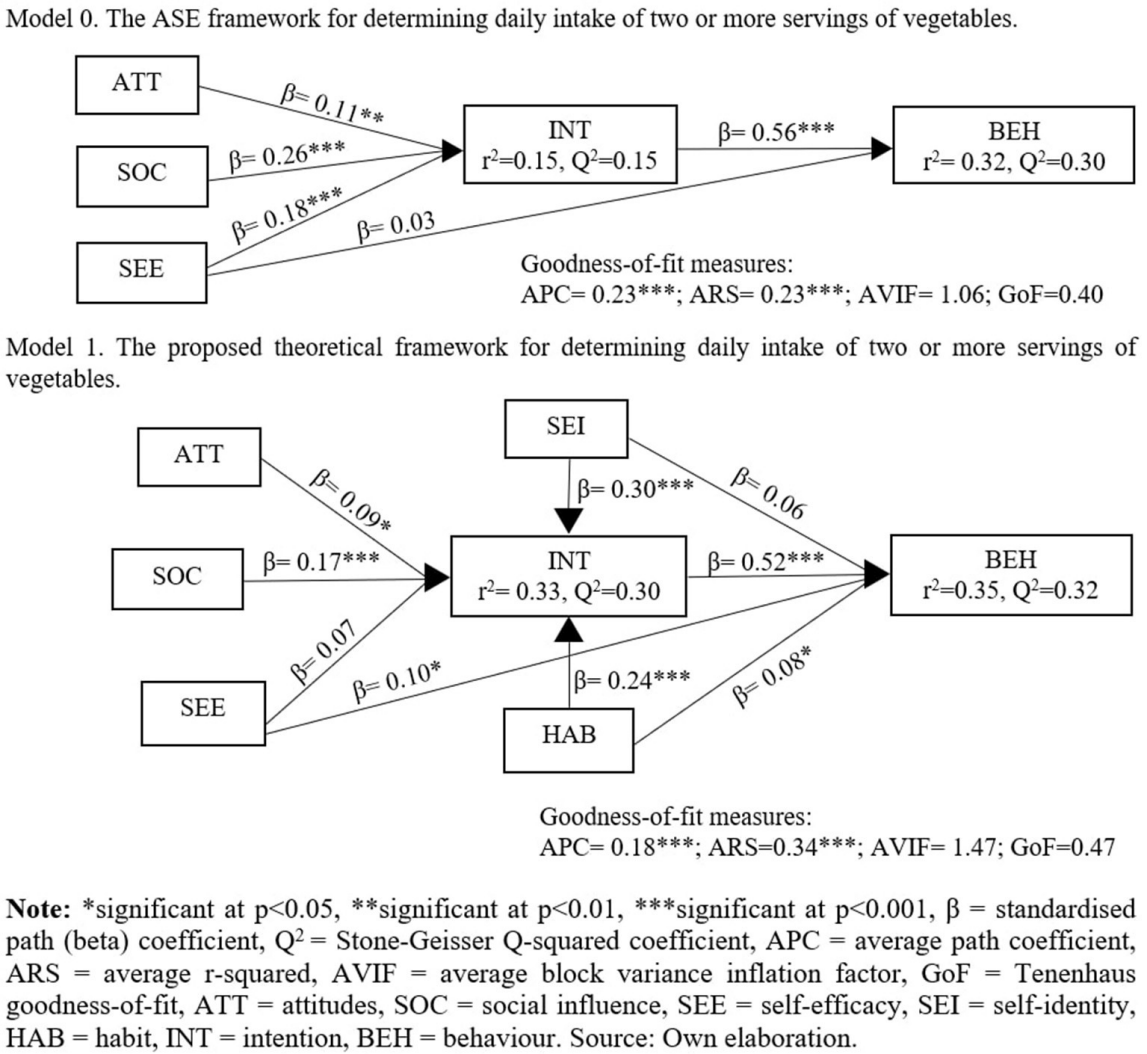
Goodness-of-fit measures.

The various path coefficients of the original ASE framework (Model 0) and the proposed theoretical framework (Model 1) are summarised in Figure 2. The results indicate that the Model 1 has a higher average r-squared (ARS) value than Model 0 (0.23 vs. 0.34, respectively), indicating that it accounted well for the variations in the behavioural intention and behaviour to eat two or more servings of vegetables per day. Thus, the proposed theoretical framework (Model 1) has the best fit with the data despite Model 0 having a higher average path coefficient (APC) than Model 1 (0.23 vs. 0.18, respectively) (44). Further, Model 1 also has a low average block variance inflation factor (AVIF), indicating no existence of multi-collinearity (44). The value of the Tenenhaus goodness-of-fit (GoF) global index of Model 0 and Model 1 are above 0.36, indicating larger effect sizes (47), however Model 1 shows a higher GoF index than Model 0 (0.47 vs. 0.40, respectively), indicating that it has the best model-data fit (48).

### Structural Model Assessment

The results from the PLSF-SEM analysis (Table 2) shows that attitudes (β = 0.09, *p* < 0.05, Cohen's *f* ^2^ = 0.02) and social influence (β = 0.17, *p* < 0.001, Cohen's *f* ^2^ = 0.05) with small to medium effect size significantly influenced intention to eat two or more servings of vegetables per day, thus hypotheses H1 and H2 are supported. In addition, self-efficacy with small effect size (Cohen's *f* ^2^ = 0.02) significantly influenced behaviour (β = 0.10, *p* < 0.05) to eat two or more servings of vegetables per day, however no significant influence was noted between self-efficacy and intention (β = 0.07, *p* = 0.06). Thus, rejecting hypothesis H3, while supporting hypothesis H4. The impact of the intention on behaviour to eat two or more servings of vegetables per day was found to be positive and significant (β = 0.52, *p* < 0.001) with a moderate to large effect size (Cohen's *f* ^2^ = 0.28), which supports hypothesis H5. In addition to the ASE constructs, habit with moderate to large effect size (β = 0.24, *p* < 0.001, Cohen's *f* ^2^ = 0.11) significantly influenced intention and a small to moderate effect size was noted between habit and behaviour to eat two or more servings of vegetables per day (β = 0.08, *p* < 0.05, Cohen's *f* ^2^ = 0.03). This supports hypotheses H6 and H7. Finally, the results also indicate that self-identity (β = 0.30, *p* < 0.001, Cohen's *f* ^2^ = 0.14) with moderate to large effect size significantly influenced intention to eat two or more servings of vegetables per day, however no significant effect (β = 0.06, *p* = 0.083) was noted between self-identity and behaviour to eat two or more servings of vegetables per day. This supports hypothesis H8, while rejecting hypothesis H9. In Model 1, the Stone-Geisser Q2 coefficient was 0.32 for intention was 0.30 for behaviour to eat two or more servings of vegetables (Figure 2), which means that the model has acceptable predictive validity (40, 50, 51), further supporting the hypotheses of this study.

**Table 2:** Structural relationship between the constructs and hypotheses' status.

**Paths**	**β-coefficient**	**Standard error**	***p*-value**	**Cohen's *f*^**2**^**	**Hypothesis**
**ATT → INT**	0.09	0.05	0.029^*^	0.02	H1: Supported
**SOC → INT**	0.17	0.05	<0.001^***^	0.05	H2: Supported
**SEE → INT**	0.07	0.05	0.062	0.02	H3: Rejected
**SEE → BEH**	0.10	0.05	0.011^*^	0.02	H4: Supported
**INT → BEH**	0.52	0.04	<0.001^***^	0.28	H5: Supported
**HAB → INT**	0.24	0.04	<0.001^***^	0.11	H6: Supported
**HAB → BEH**	0.08	0.05	0.034^*^	0.03	H7: Supported
**SEI → INT**	0.30	0.05	<0.001^***^	0.14	H8: Supported
**SEI → BEH**	0.06	0.05	0.083	0.02	H9: Rejected

* *Significant at p < 0.05*,

** *significant at p < 0.01*,

*** *significant at p < 0.001*,

*Cohen's f^2^ (49), ATT, attitude; SOC, social influence; SEE, self-efficacy; INT, intention; HAB, habit; SEI, self-identity; BEH, behaviour. Source: Own elaboration*.

Age, gender, diet, BMI, and place of residence of the participants were controlled in this study, and it was found that the diet (non-vegetarian = 1) (β = −0.10, *p* = 0.014) and place of residence (Kathmandu = 1) (β = 0.11, *p* = 0.007) of the participants had a significant impact on their daily intake of two or more servings of vegetables. In contrast, age (under 25 years = 1) (β = −0.02, *p* = 0.328), gender (β = 0.02, *p* = 0.350), and BMI (normal BMI = 1) (β = −0.04, *p* = 0.209) did not have any impact on their behaviour to eat two or more servings of vegetables per day. Further, the predictive power of vegetable consumption intention with controlled variables was *r*^2^ = 0.32 and that of vegetable consumption behaviour was *r*^2^ = 0.33.

## Discussion and Implication

This exploratory study attempts to determine psychosocial determinants influencing behaviour to eat two or more servings of vegetables per day among young adults in one of the developing countries, primarily based on the ASE framework along with habit and self-identity as additional constructs. The results of this study strongly confirm the adequacy of the ASE framework to determine recommended vegetable intake levels among Nepalese young adults. The addition of habit and self-identity in the ASE framework increased the explained variance, especially of behavioural intention. The results from the present study indicate that among the ASE constructs, social influence has a strong effect on intention to eat two or more servings of vegetables per day. These findings are inconsistent with a previous study (35). However, in Nepal, most of the young adults and their parents continue to live in the same household for multiple generations (52). Furthermore, the meals in the household are often prepared and grocery shopping is done by the mothers/guardian, therefore meal patterns and dietary intake are directly influenced by the parental knowledge of nutrition, attitude and cooking techniques (53). Furthermore, peers of similar age/partners can highly influence the behaviour positively or negatively and can provide social support (19, 54). This might explain higher social influence for intention to eat two or more servings of vegetables per day among the participants. Therefore, parental involvement intervention (55) and peer-based intervention (36) might be effective strategies to encourage vegetable consumption among young adults in Nepal.

Attitude was a significant predictor for intention to eat vegetables among young adults. The findings are consistent with previous studies of fruit and vegetable consumption among young adults (19, 56). Singh et al. (54) found that educational interventions significantly developed positive attitudes towards healthy eating behaviour among adolescents in Nepal. Such interventions might also consider providing nutritional advice on the health implications of consuming adequate vegetables for promoting and stimulating a positive attitude towards the recommended daily intake of vegetables among young adults (57).

The results from this study indicate that self-efficacy is a positive and significant predictor of behaviour to eat two or more servings of vegetables per day among young adults, however failed to significantly influence intention to eat two or more servings of vegetables per day. The findings are inconsistent with findings from previous studies (25, 58). The study (59) argued that self-efficacy appeared to be the most consistent determinant in nutrition-related interventions to successfully adopt new behaviour in Nepal. Similarly, Bhandari and Kim (60) found that self-efficacy is a strong significant predictor for health promoting behaviours among Nepalese migrant workers aged 21–55 years. Thus, psychological programmes focusing on self-efficacy enhancement through modelling, persuasion and direct mastery experience (61) is recommended. Further, such interventions might also consider including planning (action and coping) skills for achieving sustained behaviour change (62, 63), and thus to ultimately form healthy dietary habits among young adults (25).

The result from this study indicate that habit strength has a positive influence on intention and behaviour to eat two or more servings of vegetables per day. The findings are in line with previous studies (25, 30, 31). However, the study (25) found that habit strength had a stronger effect on behaviour than on intention, which is not aligned with the findings of this study. According to Verplanken and Orbell (64), intentions are expected to influence consumption of fruit and vegetables when habits are not formed as a result of occasional and irregular behaviour. Furthermore, as argued by Ouellette and Wood (65), repeated and consistent behaviour to eat fruit and vegetables is determined and gradually initiated by extrinsic stimuli. These extrinsic stimuli can be, for instance, lifestyle (66), seasonal influence (19), dietary diversity (67), parental consumption and their habits (29), availability of vegetables (68, 69), household income (70, 71), all of which may influence consumption of adequate vegetables among young adults in Nepal. In the context of Nepal, a previous study has found that low dietary diversity, low income and non-availability in households were strongly associated with consumption of insufficient fruit and vegetables (67). Further, seasonal availability strongly determined the intake of vegetables in Nepal (72, 73). Therefore, interventions to increase vegetable consumption among young adults in Nepal might include cue disruption, environmental engineering and vigilant monitoring (74), in order to prompt habitual behaviour. Previous studies have also proposed implementing intentions that can help overcome the so-called intention-behaviour gap (22, 75) to change unwanted food habits, however Rothman et al. (76) argue that such changes still require motivational efforts.

The findings from this study suggest that self-identity has a strong effect on intention to eat two or more servings of vegetables per day, however failed to significantly influence behaviour to eat two or more servings of vegetables per day. The findings align with the findings from a previous study (34). Previous studies have indicated that participants with higher self-identity are more responsive to information related to nutrition education (77, 78). The study (79) argue that health consistent behaviour can be achieved by creating healthy eating behaviour goals, and these goals for behavioural change can be fulfilled by altering them to self-as-doer identity statements. For instance, a goal can be “eat more vegetables” whereas the self-as-doer identity statement would be “vegetable eater.” Therefore, setting up goals and altering them to self-as-doer statements can increase intrinsic motivation among Nepalese young adults to eat adequate amount of vegetables (36, 80).

### Strength and Limitations

The study is the first of its kind to determine the psychosocial determinants influencing daily intake of vegetables among Nepalese young adults using the ASE as a theoretical framework with habit and self-identity as additional constructs.

The study has some limitations. First, limited sample size with cross-sectional data of young adults limits the causal inference of the findings, and only participants with English language skills and access to the internet completed the survey, thus potentially limiting the generalisation to young adults in Nepal. Further, the convenience (snowball) sampling technique employed might have resulted in the selection bias. The responses in this study were self-reported, which may have resulted in biases (self-reporting, social desirability, and recall) (81). For instance, a literature review (82) showed that bias from self-reported height and weight (underestimated and overestimated) was greater in overweight and obese participants than those of normal weight, however, such bias seemed to be lower among Asian population than those from other continents. Further, we employed a brief measure of habit strength and self-identity, and thus may have limited more precise estimations of the effects of habit strength (64) and self-identity (34). Finally, this study considered self-identity and habit as an additional construct, however inclusion of other important factors such as knowledge, perceived barriers and food consumption patterns (19, 54) could have strengthened the predictability of the framework.

## Conclusion

In conclusion, the study has empirically applied the ASE framework with two additional constructs: habit and self-identity. The additional constructs have increased the predictive ability of the proposed theoretical framework to determine the psychosocial determinants influencing daily intake of two or more servings of vegetables among Nepalese young adults. Overall, attitudes, social influence, habit and self-identity were significant factors influencing intention to eat two or more servings of vegetables per day. Further, self-efficacy and habit significantly influenced behaviour to eat two or more servings of vegetables per day. However, self-efficacy did not significantly influence intention and self-identity did not significantly influence behaviour to eat two or more servings of vegetables per day. In future, longitudinal studies with larger and statistically representative young adults are recommended. Future studies might consider investigating the effectiveness of such health behaviour models in achieving desired healthy eating behaviour among young adults in developing countries like Nepal.

## Data Availability Statement

The raw data supporting the conclusions of this article will be made available by the authors, without undue reservation.

## Ethics Statement

The studies involving human participants were reviewed and approved by Institutional Ethical Review Committee of Nepal Health Research Council. The patients/participants provided their written informed consent to participate in this study.

## Author Contributions

SP and MB did the analysis of the data, data curation, methodology, and writing the original draft. DY acted as project administrator and did supervision and validation. All of the authors participated in conceptualisation, reviewing and editing the manuscript, and gave final approval to the submitted version of manuscript.

## Conflict of Interest

The authors declare that the research was conducted in the absence of any commercial or financial relationships that could be construed as a potential conflict of interest.
