# Supplementary material for: Psychosocial Determinants of Vegetable Intake Among Nepalese Young Adults: An Exploratory Survey
Source: Front Nutr. 2021 Jun 10;8:688059. doi: 10.3389/fnut.2021.688059 (PMC8222569; doi:10.3389/fnut.2021.688059)
Supplement: Supplementary file 1 [file Data_Sheet_1.pdf]

Supplementary Material

1 Supplementary Tables

Supplementary Table 1. Items measuring the constructs of the proposed theoretical framework with their source of adoption.

Constructs	Items	Source of Adoption
Attitudes towards vegetables	ATT 1: Not pleasant – pleasant (reverse)	(2)
	ATT2: Not convenient – convenient	
	ATT3: Difficult – easy (reverse)	
	ATT4: In line – in line with my food style	
Social influence regarding vegetables	SOC1: My parents eat at least two servings of vegetables every day	(3)
	SOC2: Other close ones eat at least two servings of vegetables every day	
	SOC3: My parents encourage me to eat at least two servings of vegetables every day	
	SOC4: Other close ones encourage me to eat at least two servings of vegetables every day	
Self-efficacy to eat vegetables	SEE1: I think I can eat at least two servings of vegetables every day next week	(3,4)
	SEE2: If I decide to eat at least two servings of vegetable every day I can do it	
	SEE3: I think I could eat more vegetables when I am eating at home	
	SEE4: I think I could eat more vegetables when I am eating at a restaurant/canteen/etc	

Habit to eat vegetables	HAB1: Eating two servings of vegetables is something I do automatically	(5)
	HAB2: Eating two servings of vegetables is something I do without thinking	
	HAB3: Eating two servings of vegetables is something I do without having to consciously remember it	
Self-identity	SEI1: I think of myself as a healthy eater	(6)
	SEI2: I think of myself as a person who is interested in eating healthy foods	
	SEI3: I think of myself as someone who is concerned about the health consequences of what I eat	
Intention to eat two or more servings of vegetables per day	INT1: I intend to eat at least two servings of vegetables per day next week	(2)
	INT2: I am sure to eat at least two servings of vegetables per day next week	
Behaviour to eat two or more servings of vegetables per day	BEH1: Number of servings of vegetables items participants have eaten during the last 24 hours	(2)
	BEH2: Number of servings of vegetables participants have eaten last week	

Supplementary Table 2. Factor loadings, validity, reliability and multicollinearity tests of the proposed theoretical framework.

Constructs	Items	Factor loadings	Cronbach alpha	AVE	CRC	VIF
**Attitude towards vegetables**	ATT1	0.97	0.94	0.75	0.92	1.05
	ATT2	0.99				
	ATT3	0.79				
	ATT4	0.96				
**Social influence regarding vegetables**	SOC1	0.87	0.82	0.51	0.80	1.15
	SOC2	0.89				
	SOC3	0.93				
	SOC4	0.91				
**Self-efficacy to eat vegetables**	SEE1	0.83	0.87	0.61	0.86	1.53
	SEE2	0.77				
	SEE3	0.79				
	SEE4	0.76				
**Habit to eat vegetables**	HAB1	0.73	0.79	0.57	0.79	1.74
	HAB2	0.70				
	HAB3	0.77				
**Self-identity**	SEI1	0.72	0.87	0.70	0.87	1.95
	SEI2	0.70				
	SEI3	0.78				

**Intention to eat two or more servings of vegetables per day**	INT1	0.77	0.91	0.84	0.91	1.86
	INT2	0.76				
**Behaviour to eat two or more servings of vegetables per day**	BEH1	0.80	0.75	0.60	0.75	1.50
	BEH2	0.85				

Note: Each factor loadings were normalised and were significant at p <0.05 in their corresponding construct, AVE = average variance extracted, CRC = composite reliability, VIF = variance inflation factor.

Supplementary Table 3. Descriptive statistics and correlation coefficients between the constructs.

**Constructs**	**ATT**	**SOC**	**SEE**	**HAB**	**SEI**	**INT**	**BEH**[a]
**Attitude (ATT)**	**0.86**						
**Social influence (SOC)**	0.12**	**0.71**					
**Self-efficacy (SEE)**	0.02	0.12**	**0.78**				
**Habit (HAB)**	0.06	0.27***	0.46***	**0.75**			
**Self-identity (SEI)**	0.02	0.18***	0.56***	0.57***	**0.83**		
**Intention (INT)**	0.14**	0.28***	0.23***	0.44***	0.44***	**0.91**	
**Behaviour (BEH)**	0.18***	0.07	0.14**	0.31***	0.22***	0.55***	**0.77**
**Mean**	4.48	5.73	5.52	5.05	5.37	5.39	61.6%
**Standard deviation**	1.63	1.06	1.02	1.27	1.06	1.10	(284)

Note: **significant at p <0.01, ***significant at p <0.001 (two-tailed); bold numbers are the square root values of average variance extracted (AVE), [a]percentage (%) and proportion (in brackets), proportion and percentage were calculated for the dichotomous variable Behaviour (BEH), correlation coefficient were estimated employing Pearson's correlation tests.
